# Predictors for choosing the specialty of Family Medicine from undergraduate knowledge and attitudes: Preditores da escolha da especialidade Medicina de Família a partir de competências e atitudes de alunos de graduação

**DOI:** 10.1590/1516-3180.2015.02581002

**Published:** 2016-06-27

**Authors:** María Candelaria Ayuso-Raya, Francisco Escobar-Rabadán, Jesús López-Torres-Hidalgo, Julio Montoya-Fernández, Juan Manuel Téllez-Lapeira, Francisco Campa-Valera

**Affiliations:** I MD. Family Physician at the Emergency Service of Albacete General Hospital, Healthcare Service of Castilla-La Mancha (SESCAM), Albacete, Spain.; II MD. Family Physician at the Zone 4 Healthcare Centre, SESCAM, Albacete, Spain.; III MD. Associate Professor of the Albacete Medical School and Family Physician at the Zone 8 Healthcare Centre, SESCAM, Albacete, Spain.; IV MD. Deputy Chief Medical Officer for Primary Healthcare, SESCAM, Albacete, Spain.; V MD. Associate Professor of the Albacete Medical School and Family Physician at the Zone 5b Healthcare Centre, SESCAM, Albacete, Spain.; VI MD. Associate Professor of the Seville Medical School and Family Physician at the Don Paulino García Donas Healthcare Centre, Healthcare Service of Andalucía (SAS), Andalucía, Spain.

**Keywords:** Family practice, Health knowledge, attitudes, practice, Medical education, graduate, Primary health care, Students, medical, Medicina de família e comunidade, Conhecimentos, atitudes e prática em saúde, Educação de pós-graduação em medicina, Atenção primária à saúde, Estudantes de medicina

## Abstract

**CONTEXT AND OBJECTIVE:**

A cold climate towards primary care (PC) within medical academia could form a barrier against choosing family medicine (FM) as a career option. This study was designed to determine whether medical students’ knowledge of and attitudes towards FM predicted their career choice.

**DESIGN AND SETTING:**

Cohort study conducted at two different medical schools.

**METHODS:**

After completing a PC course at the Albacete Medical School in 2005-2006, 81 second-year students were asked to give responses to a questionnaire. In their sixth year (2009-2010), 79 students in Albacete and 42 in Seville (taken as an unexposed cohort) were asked to give responses too. Their choice of specialty was investigated in 2011.

**RESULTS:**

In Albacete, the questionnaire was answered by 79 second-year and 76 sixth-year students; in Seville, it was answered by 26 sixth-year students. After completing the PC course, 69.3% said they would like to become a family doctor. This percentage decreased to 40.3% at the end of the undergraduate course (P < 0.0001). In the sixth year, the attitudes towards FM worsened, yet these were significantly more favorable than those in Seville. Only 12 students chose FM; they obtained significantly worse scores in their specialty selection examination than their peers (P < 0.0001).

**CONCLUSION:**

In the Albacete Medical School, the students’ opinion about FM worsened over the undergraduate course, although it was still better than the Seville students’ stance. In any case, FM was seen to be a minority option.

## RESUMO

**CONTEXTO E OBJETIVO:**

Um clima frio para a atenção primária na academia médica constitui uma barreira para escolher Medicina de Família (MF) como opção de carreira. Este estudo foi concebido para determinar se o conhecimento e as atitudes dos estudantes de medicina em relação à MF predizem a escolha da carreira.

**TIPO DE ESTUDO E LOCAL:**

Estudo de coorte realizado em duas faculdades de medicina.

**MÉTODOS:**

Depois de terem completado um curso de Cuidados Primários na Faculdade de Medicina de Albacete, em 2005-2006, 81 alunos do segundo ano foram convidados a responder a um questionário. No seu sexto ano (2009-2010), 79 estudantes de Albacete assim como 42 de Sevilha, tomados como coorte não exposta, foram convidados a responder também. Todos eles foram investigados sobre a escolha da especialidade em 2011.

**RESULTADOS:**

Em Albacete, 79 e 76 estudantes responderam no segundo e sexto anos, respectivamente, e 26 em Sevilha. Depois de terem concluído o curso de cuidados primários, 69,3% disseram que gostariam de se tornar médicos de família. Esta percentagem diminuiu para 40,3% no final da graduação (P < 0,0001). No sexto ano, as atitudes com relação à MF pioraram, mas estas foram significativamente mais favoráveis do que as de Sevilla. Apenas 12 alunos escolheram a MF; eles obtiveram pontuação significativamente piores no exame do que seus pares (P < 0,0001).

**CONCLUSÃO:**

Na Faculdade de Medicina de Albacete, a opinião dos alunos sobre a MF ao longo da graduação piorou; contudo ainda era melhor que as dos estudantes de Sevilha. Em qualquer caso, MF foi opção minoritária.

## INTRODUCTION

For many years, a primary care (PC) physician shortage has been highlighted worldwide. This very likely contributes towards fragmented care, inappropriate use of specialists and less emphasis on prevention. The reasons for this are multifold. First of all, the interest in a career in PC has declined over recent years, and the number of medical school graduates selecting a family medicine (FM) career has declined markedly.^1^^,^^2^^,^^3^ According to the Association of American Medical Colleges (AAMC), these numbers can be explained by an unfavorable practice environment within PC (i.e. poor quality working conditions due to high patient loads, and public and private reimbursement systems that undervalue PC services in comparison with procedures performed by specialists), as well as by perceptions of status and prestige. Also according to the AAMC, medical education and training have a lesser impact on career choices.^4^

However, a cold climate towards PC has traditionally been recognized within medical academia. This could constitute a barrier against choosing this discipline as a career option.^5^

In a previous study, we determined that students at the Albacete Medical School showed a noteworthy initial lack of knowledge and a poor opinion of FM and PC. We also demonstrated that changes in the knowledge of and attitudes towards FM took place after these students completed a course on PC.^6^ In short, student’s experiences during clinical clerkships or while undertaking specific courses with a field of medicine have a significant impact on their attitudes and interest in a certain specialty afterwards.^7^^,^^8^^,^^9^ Especially, it has been pointed out that PC training at preclinical stages contributes towards better clinical performance, because it can help medical students to acquire the fundamental cognitive and clinical skills that they will apply during the clinical years of medical training.^10^ These clerkships can have a long-term positive effect on approaches towards FM.^11^

FM has existed as a specialty in Spain for more than 30 years. Nevertheless, FM training in Spain has only recently been provided within university courses and its provision around the country remains uneven. Spanish medical schools’ offer of FM training ranges from absence from the study program to provision of clinical clerkships alone (either on a mandatory or on an elective basis) or inclusion of FM as a mandatory subject.

Until the European Union higher education reform known as the Bologna Process, the Albacete Medical School (at that time about a decade old) had a mandatory subject on PC for medical students in the second year of a six-year undergraduate program. That used to be the students’ first clinical experience. The PC course was four months long and provided five credits (such that one credit represented 10 hours of training). Out of those five credits, 1.5 were theory credits. The rest were practical credits: students were required to complete a one-week clinical clerkship in a PC center within the city of Albacete. During that week, students accompanied a family doctor during all of his or her daily activities. Now, due to the change in the study program, FM is taught in the fifth year. At Seville Medical School, students were taking a mandatory FM subject in their sixth year at the time when this study was conducted.

That clerkship on PC was our students’ first clinical experience and this could, in the words of Miettola et al., have caused a honeymoon effect.^12^ Thus, we need to consider how students’ opinions on FM and PC will develop over their future academic years. The students’ positive perceptions about PC practice may change as realistic perceptions about the professional demands on PC doctors arise during medical school, as Cooter et al. pointed out.^13^ However, there is a possibility that this attitude towards FM may become even more positive when medical students end their undergraduate years. This might be partially explained by greater contact with family doctors. Regarding the latter, it should be emphasized that the Albacete Medical School students had, besides the clerkship on PC in the second year (i.e. the one discussed here), another training week within the third year (on Psychology in the Clinical Setting) and another one in their fifth year (within Medicine and Surgery II).

For these reasons, we always stressed that there was a need for our study to continue with a further assessment of senior students who are close to getting their degrees, as well as an investigation of their specialty choice.

## OBJECTIVE

Our hypothesis was that taking a course in PC not only would improve students’ knowledge of FM and help them to develop a more positive attitude towards it, but also would lead those with more favorable attitudes to be more likely to choose this specialty. Our goal was to determine whether medical students’ knowledge of and attitudes towards FM would change between the second and sixth year, and whether these would predict selection of FM as their specialty.

## METHODS

We conducted a cohort study among Albacete Medical School students who took a PC subject in their second year and used Seville Medical School students (before they started their school’s FM course in their sixth year) as an unexposed cohort.

### Study variables

The dependent variable was the result from applying the original version of the Valuation of Attitudes towards and Knowledge of Family Medicine Questionnaire (CAMF, in the Spanish acronym).^14^ This is formed by 34 closed-response items (for example: “I would like to become a family doctor in the future”), with five response options on a Likert scale. The questionnaire also contains items on the sociodemographic and academic characteristics of the students: age, sex, number of inhabitants in the city/town that they come from, social class estimated according to the Domingo and Marcos classification (based on parents’ occupation),^15^ number of subjects still pending and “grades from medical school entrance examinations (which depend on the university access test and high school average marks).

### Study procedures

On the day of the final examination, the Albacete students taking the PC subject in the 2005-2006 school year were asked to give responses to a self-administered, anonymous questionnaire. The students were again invited to answer the same questionnaire at the end of their degree course, i.e. when they were final-year students in 2009-2010. In the same school year, final-year students who had been enrolled in an FM subject at the Seville Medical School (Valme campus), were invited to join in the study as an unexposed group, before they started to attend this course.

We registered the specialty that these students chose after their specialist medical training access examination (MIR, in the Spanish acronym) equivalent to residency in 2011, based on the information provided by the Spanish Ministry of Health on its website.

The data gathered were coded and entered into a computerized database using the SPSS 19.0 statistical software.

### Ethical aspects

Ethical approval for this study was granted by the Investigational and Clinical Ethics Committee of the Albacete Health Area.

Student participation in the study was voluntary. In order to compare related samples when needed, the students were asked for a reference number. It was agreed between the students and the research group that the former would sign their answer sheets with the last four digits of their National Identity Card, since this would be easier for them to remember. In any case, we guaranteed the anonymity of their responses.

### Statistical analysis

We analyzed the responses to the items and calculated the overall score of the questionnaire, giving the following values: “completely disagree”: -2; “disagree”: -1; “indifferent”: 0; “agree”: +1; and “completely agree”: +2. In order to make the “-2” value always correspond to the most unfavorable option regarding FM and +2 to the most favorable, we recoded the responses to items 15, 22, 23, 25 and 26 with inverted scales.

The statistical analysis included a description of the different variables, comparing the groups of students. We analyzed the responses to the 34 questionnaire items, thus evaluating Albacete students’ level of knowledge and their attitudes at the end of the second and sixth school years. We used the Wilcoxon signed-rank sum test to evaluate the statistical significance of the possible changes in scores for the different items. We also calculated the effect size for each item.^16^ The questionnaire responses of the Albacete students were compared with those of the students in Seville using the Mann-Whitney test. Moreover, we calculated the effect size for each item.

A comparative analysis on the responses according to the different sociodemographic and academic variables was made using Pearson’s chi-square test to compare proportions in independent groups. Student’s t test was applied to compare the means of normally distributed continuous variables, while the nonparametric Mann-Whitney test was used in other situations. The mean score from the questionnaire was compared for different values of the sociodemographic and academic variables, using Student’s t test in the case of dichotomous variables and Pearson’s linear correlation in the case of continuous variables. Multiple linear regression analysis was also used. Logistic regression was used to determine the association between choosing FM and other conditioning factors, and to avoid possible confounding variables.

### RESULTS

In Albacete, the questionnaire was completed by 79 undergraduates at the end of their second year (97.5% of the total number enrolled) and by 76 undergraduates (96.2%) at the end of their sixth year; 62 students completed the questionnaire on both occasions. Meanwhile, at the Seville Medical School, 26 students (61.9% of those enrolled) completed the questionnaire.

Table 1 sets out the sociodemographic and academic characteristics of the students who participated in the study. There were some differences between the groups: the proportion of women was significantly higher in the Seville Medical School (84.6%) and there were more students coming from small towns; the Albacete students had higher grades on entry to the medical school and a higher proportion of them did not have any pending subjects. We did not find any statistically significant differences regarding age or social class.

**Table 1.**
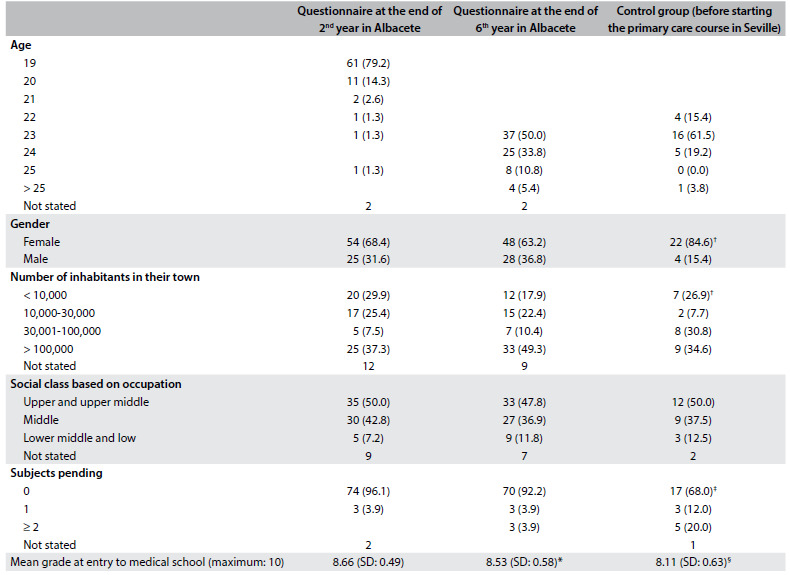
Sociodemographic and academic characteristics of the students participating in the study (in brackets: % in relation to total who responded)

*P = 0.001; ^†^P = 0.04; ^‡^P = 0.007; ^§^P = 0.003; SD = standard deviation.

Table 2 presents the median and the interquartile range for each CAMF item in the second and sixth years for the students at the Albacete Medical School and in the sixth year for the Seville students. Tables 3 and 4 show items with statistically significant differences: items relating to knowledge of PC and FM and items relating to a positive attitude towards the work of family doctors, respectively. Table 5 sets out items showing statistically significant differences between sixth-year students in Albacete and Seville.

**Table 2.**
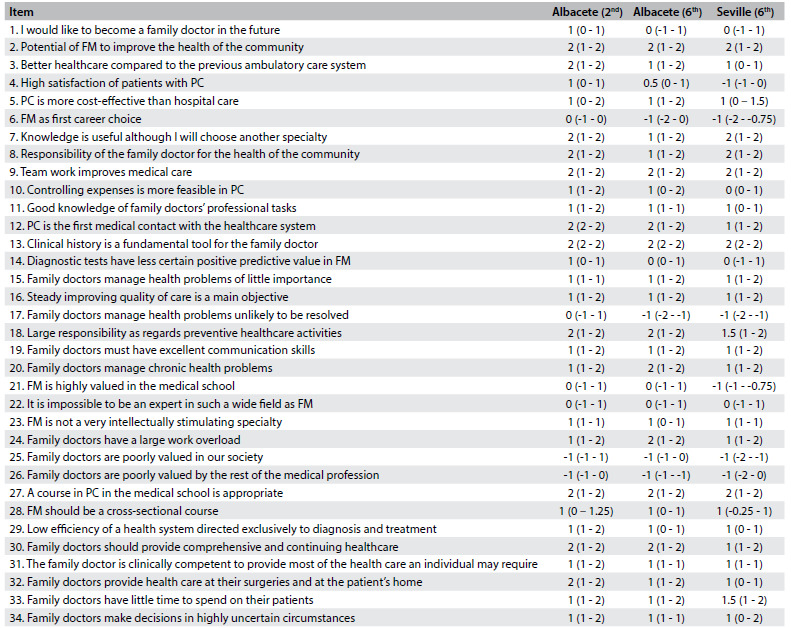
Median (with interquartile range) for each of the items of the Valuation of Attitudes towards and Knowledge of Family Medicine Questionnaire (CAMF).

FM = family medicine; PC = primary care.

**Table 3.**
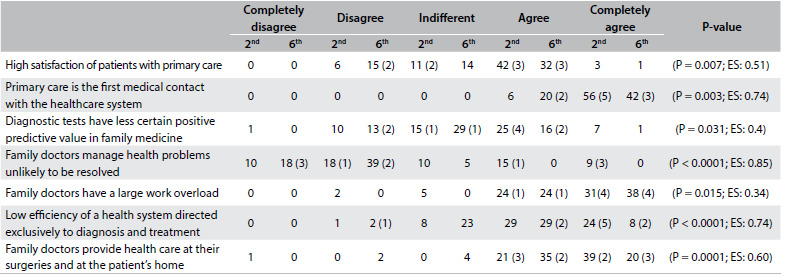
Albacete 2^nd^ versus 6^th year^: items relating to knowledge of primary care and family medicine that showed significant differences.

EF = effect size. In brackets, the number of graduates choosing family medicine in each rank.

**Table 4.**
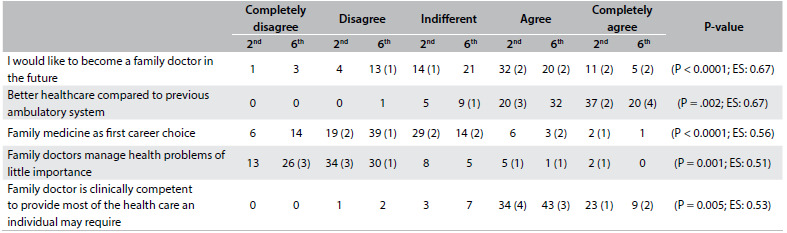
Albacete 2^nd^ versus 6^th^ items relating to attitudes towards primary care and family medicine that showed significant differences.

EF = effect size. In brackets, the number of graduates choosing family medicine in each rank.

**Table 5.**
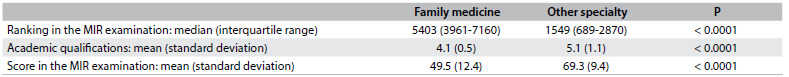
Ranking* obtained, mean of academic qualifications valued as certificated merits and mean score on the specialist medical training access examination (MIR, in the Spanish acronym), for those who chose family medicine and those who chose other specialties

*The MIR candidates are ranked according to their final score and choose their specialty following that order (i.e. the first-placed candidate, with the highest score, will be the first to choose).

After completing the PC course, 69.3% of the students said that they would like to become a family doctor in the future. This percentage decreased to 40.3% at the end of the degree course (P < 0.0001), without statistical differences in relation to the students in Seville. Five medical school graduates from Albacete chose FM. One of them had previously showed disagreement when answering the item “I would like to become a family doctor in the future” in the sixth grade. In Albacete, 12.9% of the students considered FM to be their first career choice at the end of the PC course, yet this percentage halved at the end of the degree course.

In the sixth year, a lower level of agreement with statements that could be considered more favorable towards FM and PC was generally observed, in relation to both knowledge and attitudes. Whereas there were higher levels of agreement with “Family doctors have a large work overload”, there were also higher levels of disagreement with “Family doctors manage health problems of little importance” and with the statement that these problems were “unlikely to be resolved”.

Despite the worsening stance towards PC and FM among Albacete students, their attitudes remained significantly more favorable than those of students in Seville. Nevertheless, the latter had a remarkably higher level of agreement regarding the item “Knowledge of FM is useful although I will choose another specialty”.

We compared mean CAMF scores between the second and sixth years among 57 students with full data: 33.8 (standard deviation, SD: 9.2) and 28.5 (SD: 7.1), respectively (P < 0.0001). No relationship was found within the mean difference in CAMF scores between the second and sixth years for any of the variables analyzed. The mean CAMF score could be calculated for 72 sixth-year students in Albacete and 24 in Seville: 28.4 (SD: 7.3) and 22.6 (SD: 6.0), respectively (P = 0.001).

The graduates’ preferred specialty was known in 104 cases: 72 in Albacete and 32 in Seville respectively. Twelve (five in Albacete and seven in Seville) chose FM after the MIR examination. Postgraduates choose their specialty based on the score achieved: the candidate who has the best score is ranked as number 1 and has first choice, and so on. As can be seen in Table 6, the students who chose FM obtained significantly worse scores in the examination than those who chose other specialties. There were also statistically significant differences in the means of academic qualifications that were taken into consideration for MIR and in the mean score from MIR. Both the examination scores and the qualifications were significantly higher among those who chose other specialties rather than FM. There were no significant differences relating to access grades for medical school, age, sex, social class, medical school, number of inhabitants in their town of origin or the city where they chose to do their residency.

**Table 6.**
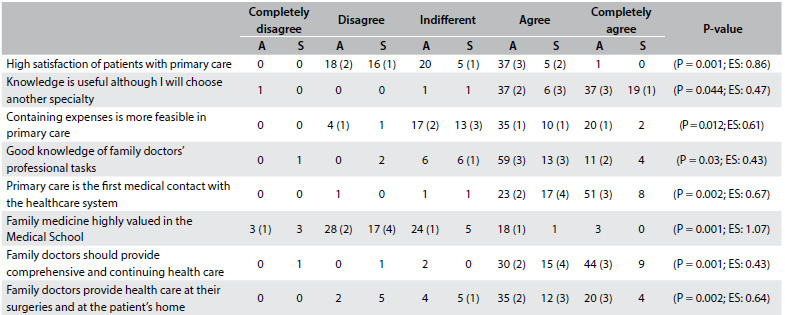
Albacete (A) 6^th^ versus Seville (S) 6^th^ year items that showed significant differences.

EF = effect size. In brackets, the number of graduates choosing family medicine in each rank.

The logistic regression analysis showed an independent association for the choice of FM only with the ranking in the MIR examination (OR: 1.001; 95% CI: 1.001-1.002).

### DISCUSSION

In the Albacete Medical School, the students’ opinions about FM and PC declined over the degree course, although they remained higher than those of the students in Seville before the latter started their course on FM. In any case, FM was seen to be a minority option as a specialty, with no significant differences between the two medical schools.

Medical career choice is complex and multifactorial.^17^ A well-known theoretical model for medical students’ specialty choice that was developed some years ago identified three components: factors associated with students’ own features, type of school and students’ perceptions of the medical specialty characteristics.^18^ As pointed out in our introduction, there is a fairly widespread belief that students’ experiences during clinical clerkships or specific courses have a significant impact on their attitudes towards specialties.^7^^,^^8^^,^^9^^,^^19^^,^^20^ PC training mostly takes place at the end of the degree course. However, at the Albacete Medical School, the PC subject was taught in the second year. Other medical schools include PC training at preclinical stages, and it has been demonstrated that such training has contributed towards better clinical performance by the students.^10^ Early experience could motivate and satisfy undergraduates, help them acclimatize to clinical environments, develop professionally, interact with patients with more confidence and less stress, develop self-reflection and appraisal skills, and develop a professional identity. It could also strengthen their learning and make it more real and relevant to clinical practice.^21^ Nevertheless, the present study showed that the students’ positive perceptions about PC services at the end of the second year may change, maybe because realistic perceptions about the demands on PC doctors end up being disseminated among undergraduates as they pursue their degrees.^13^

The study by Xu et al. may clarify this issue.^22^ They asked general practitioners in the USA whether they had any strong interest in PC before medical school and whether their level of interest changed during medical school, with special regard to their clinical experiences of this type of care. They found that for 7%, the level of interest in PC decreased during their undergraduate training; for 48%, it remained constant; and for 45%; it increased. Increased interest in PC was strongly associated with having taken elective PC courses during medical school. However, clinical experiences of PC had no impact on students’ interest in pursuing PC specialties. Therefore, students choosing a curriculum consistent with their expectations and prior inclinations would be the ones who might display increasing interest in a general practice career. Furthermore, those whose interest increased during their undergraduate training, compared with those with declining attention to PC, would be more likely to remain in PC specialties ten years after graduation. This is another indicator of the importance of medical education, not only for increasing interest in PC but also for maintaining it after graduation.

Unlike what is stated in the present paper, Martín Zurro et al. found that interest in FM increased moderately over the years of study.^23^^,^^24^ These results must be assessed with caution, since the study by Martín Zurro was cross-sectional and therefore lacks the added value achieved in the present study through following a group of classmates from second to sixth year and until choosing their specialty. They collected opinions from first, third and fifth-year students in 22 medical schools in Spain during the first quarter of the 2009-2010 and 2011-2012 academic years. The appeal of FM increased over the years of study (36.7%, 41.7% and 50.2% in years one, three and five respectively; P < 0.001), irrespective of student profile or medical school attended. Among third and fifth-year students, 54.6% said that their specialty preferences had changed over their time at medical school.

As these authors stressed elsewhere,^24^ although some students generally find FM appealing, it is regarded as a career of low interest and prestige. These authors suggested seven broad themes to explain this situation: the scope and context of practice (the perception that FM is a varied specialty, with broad practice, holistic perspective and flexibility that allows practitioners to have a family); work of lower interest or that is intellectually less challenging (treating common disease, repetitive work and almost an administrative job); influence of role models, either positive or negative, and of society (negative comments from other professionals, peers and family); lower prestige; poor remuneration; medical school influences; and postgraduate training, where conversely the shorter duration and the lower intensity were perceived as positive aspects of FM.^25^

López-Roig et al. agreed with this description of the scope. In their view, FM appears to be largely underestimated as a professional activity among medical undergraduates, perceived as monotonous and non-technological medical practice with no intellectual challenge.^26^ Such a negative point of view, which already appears in the early stages of medical training, leads to lack of identification with this medical practice among students.

Although from our previous experience^27^ we had considered that female students, especially young female students, would express a more favorable attitude towards FM and PC, the results from this present study do not confirm this statement. Other factors must undoubtedly play a role in choosing a specialty. It has been suggested that the working conditions in FM have a decisive influence on selecting this specialty,^23^ and also that the remuneration mechanism has a selection effect on new graduates who would like to become general practitioners.^28^ Lifestyle-related factors are probably equally important for men and women.^29^^,^^30^ An awareness that general practice is a flexible option may be important; although embracing this as a motivation in choosing FM as a career may occur at the expense of real interest, enthusiasm and vocation, thus risking the sustainability of FM. Other specialties are increasing the availability of flexible training and work, thus contributing towards a continuing trend of women rejecting general practice in favor of other specialties. After removing the influence of lifestyle factors and flexibility, women are probably not more likely to choose general practice than men. This opens up a broad line of research.

Our study has some limitations. The fact that PC teachers handed out the questionnaire to students might have biased the study through having a positive influence on the answers. Another possible limitation of this study is the fact that the questionnaires were applied immediately after the class’s examination, which may have led students to respond more positively than they would really have done in other situations. We were aware of this potential limitation, but we took this path because of feasibility issues.

The manner of selecting the unexposed cohort group was a matter of debate. We chose students from a medical school in which the PC course is taught in the sixth year. We could have chosen another school, in which this subject was not taught. However, we preferred the first option for two reasons: first, students at a school in which PC is not taught may show very obvious differences in relation to our students; and secondly, we wanted to test and compare the influence of a PC course in the early and final years.

### CONCLUSION

In the Albacete Medical School, students’ opinions on FM declined over the degree course, although they remained higher than those of the students in Seville. In any case, FM was seen to be a minority option as a specialty.
